# The first characterization of airborne cyanobacteria and microalgae in the Adriatic Sea region

**DOI:** 10.1371/journal.pone.0238808

**Published:** 2020-09-10

**Authors:** Kinga A. Wiśniewska, Sylwia Śliwińska-Wilczewska, Anita U. Lewandowska

**Affiliations:** 1 Division of Marine Chemistry and Environmental Protection, Institute of Oceanography, University of Gdansk, Gdynia, Pomerania, Poland; 2 Division of Marine Ecosystems Functioning, Institute of Oceanography, University of Gdansk, Gdynia, Pomerania, Poland; Kaohsiung Medical University, TAIWAN

## Abstract

The presence of airborne cyanobacteria and microalgae as well as their negative impacts on human health have been documented by many researchers worldwide. However, studies on cyanobacteria and microalgae are few compared with those on bacteria and viruses. Research is especially lacking on the presence and taxonomic composition of cyanobacteria and microalgae near economically important water bodies with much tourism, such as the Adriatic Sea region. Here, we present the first characterization of the airborne cyanobacteria and microalgae in this area. Sampling conducted between 11^th^ and 15^th^ June 2017 revealed a total of 15 taxa of airborne cyanobacteria and microalgae. Inhalation of many of the detected taxa, including *Synechocystis* sp., *Synechococcus* sp., *Bracteacoccus* sp., *Chlorella* sp., *Chlorococcum* sp., *Stichococcus* sp., and *Amphora* sp., poses potential threats to human health. Aside from two green algae, all identified organisms were capable of producing harmful metabolites, including toxins. Moreover, we documented the presence of the cyanobacterium *Snowella* sp. and the green alga *Tetrastrum* sp., taxa that had not been previously documented in the atmosphere by other researchers. Our study shows that the Adriatic Sea region seems to be a productive location for future research on airborne cyanobacteria and microalgae in the context of their impacts on human health, especially during the peak of tourism activity.

## Introduction

Bioaerosols comprise living and dead organisms as well as their fragments and excrements emitted from the biosphere into the atmosphere [1–3]. Bioaerosols include archaea, fungi, microalgae, cyanobacteria, bacteria, viruses, plant cell debris, and pollen [1–5]. The most poorly studied organisms in aerobiology and phycology are airborne microalgae and cyanobacteria [4, 6].

This lack of knowledge may result from the lack of standard methods for both sampling and further analysis, especially quantitative analytical methods [5]. Few studies have been performed to determine the number of cyanobacteria and microalgae in the atmosphere [7, 8]. A previous review [2] has shown that the average quantity of atmospheric algae is between 100 and 1000 cells per cubic meter of air. Currently, over 350 taxa of cyanobacteria and microalgae have been documented in the atmosphere worldwide [5, 9]. Cyanobacteria and microalgae end up in the air as a consequence of their emission from soil, buildings, trees and roofs [5, 10, 11].

The environmental role of airborne cyanobacteria and microalgae is only partly understood. While present in the air, cyanobacteria and microalgae can contribute to ice nucleation and cloud droplet formation. Cyanobacteria and microalgae can also impact human health [2, 5, 9, 12–14]. Depending on their size, airborne cyanobacteria and microalgae can be inhaled by humans and settle in different parts of the respiratory system, leading to the formation or intensification of numerous diseases and ailments, e.g., allergies, dermatitis, and rhinitis [9, 15, 16]. According to Wiśniewska et al. [5], these harmful microorganisms can constitute between 13% and 71% of sampled taxa.

Research on airborne algae is especially important in tourist areas near water-bodies. Sunbathers are exposed to particularly high quantities of harmful cyanobacteria and microalgae. Additionally, harmful microalgae and cyanobacteria blooms tend to occur in both marine and freshwater reservoirs during summer [17–21]. Previous work has shown that the Mediterranean Sea is dominated by the picocyanobacteria *Synechococcus* sp. and *Synechocystis* sp., which are responsible for the production of a group of hepatotoxins known as microcystins [22]. Our previous work has also shown that these species can be found in aerosols in the Adriatic Sea. Because most tourism occurs in summer, many tourists are exposed to the most extreme negative impacts of airborne microalgae.

The main goal of our study was to identify the cyanobacteria and microalgae in the atmosphere of the northern Mediterranean Sea region (specifically, the Adriatic Sea) due to its tourist advantages and economic importance for Europe and rest of the world. In addition, our study provides the first information on the percentage of individual taxa in the atmosphere in this region. This preliminary study demonstrates the need for more research on airborne cyanobacteria and microalgae in the northern Mediterranean Sea region.

## Materials and methods

**Fig 1:**
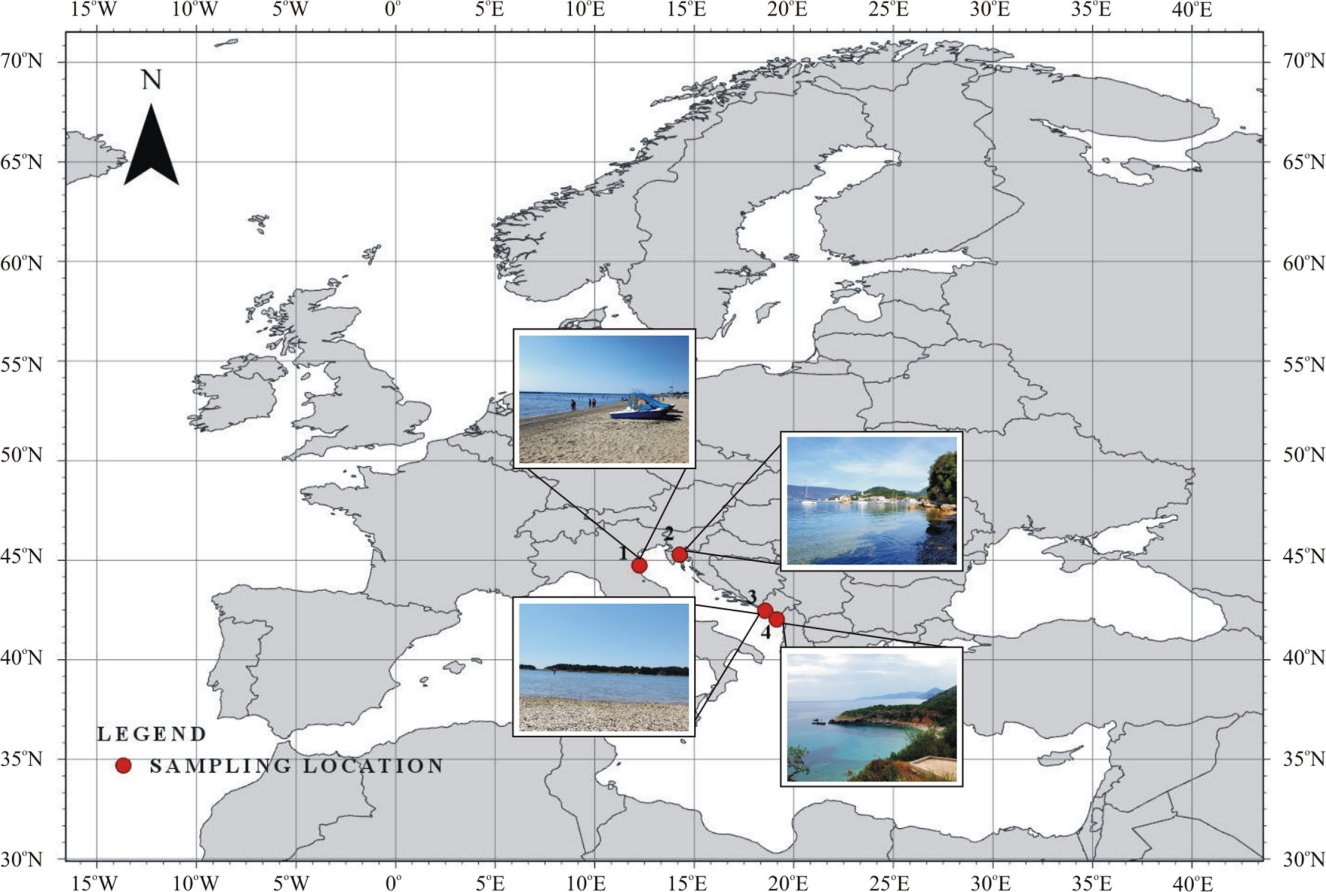
Map of airborne algae and cyanobacteria sampling locations in the northern Mediterranean Sea region– 1^st^ station in Italy, 2^nd^ station in Croatia, 3^rd^ and 4^th^ in Montenegro (ArcGIS PRO 2.3.2).

Samples of airborne microalgae and cyanobacteria were collected from four stations over the Mediterranean Sea (specifically, the Adriatic Sea) during the high tourist season, between the 11^th^ and 15^th^ of June 2017. The locations of the measurement stations, country of origin, times of sample collection, and environmental conditions during the sampling period are shown in S1 Table. One research station was located in the northern part of Italy (station 1), one in Croatia (station 2), and two in Montenegro (station 3 and station 4). These research stations were strategically placed to estimate the differences in the qualitative compositions of airborne cyanobacteria and microalgae in disparate regions of tourism in the Adriatic Sea. All the sampling stations were located on beaches at a distance of 50 m from the seashore (Fig 1).

Prior to collection, a sterile mineral f/2 culture medium was prepared [23] and calibrated using sea water with a salinity of 32 PSU. Petri dishes with liquid f/2 medium (V = 5mL) were exposed for 1 h to collect deposited material [24, 25]. The use of a liquid medium facilitated material transfer and also limited the growth of bacteria and fungi, which often prefer to grow on agar [26]. Indeed, bacteria and fungi may overgrow cyanobacteria and microalgae in samples [5, 27]. At each site, three Petri dishes were fastened at a height of 1 m above the ground. After collection, airborne algae were cultured for 3 weeks in incubators under constant conditions of 20°C and a 16:8 h light:dark cycle at 10 μmol photons m^–2^s^–1^. Fluorescent lamps (Cool White 40W, Sylvania, Wilmington, Massachusetts, USA) were used as a source of irradiance, and the photosynthetically active radiation (PAR) intensity was measured with a quantum-meter (LI-COR; Lincoln, Nebraska, USA).

Morphological analysis of airborne microalgae and cyanobacteria was conducted under a light microscope (Nikon Eclipse 80i; Tokyo, Japan) at magnifications of 10x and 100x. Additionally, an epifluorescence microscope (Nikon Eclipse 80i; Tokyo, Japan) with a green excitation/block filter (EX 510–560, DM 575, BA 590, 6-2A) was used to verify the collected material [16]. Collected organisms were identified to the lowest possible taxonomic level. Identification of airborne microalgae and cyanobacteria was conducted with the aid of taxonomic keys and the literature [e.g., 28–35].

Meteorological parameters were downloaded from OGIMET (http://www.ogimet.com), and the air mass 48 h backward trajectory was obtained using the HYSPLIT model for each day, using 6-h intervals (Hybrid Single-Particle Lagrangian Integrated Trajectory) [36, 37]. HYSPLIT is a complete system for computing simple air parcel trajectories to complex dispersion and deposition simulations [36, 37]. To determine the direction of air mass overlap the three standard air mass arrival heights (500, 1000 and 1500 meters AGL) were used [1]. This information helps to determine the source of both chemical and biological air pollution by tracking the path of air masses at a given time prior to sampling [1, 16, 25]. The starting height was always set at the collection height, 1 m AGL, in order to interpret our results.

All the figures containing maps have been done in ArcGIS PRO 2.3.2 on which we have advanced license. The license is provided by the University of Gdansk. To obtain a consistent database, authors determined taxa from algal and cyanobacterial species. Based on the above data using the ArcGIS PRO 2.3.2 software by ESRI, authors determined the locations (points) where the research was carried out. Maps showing points and pie charts were created in the ArcGIS PRO 2.3.2 software. All images were made by authors of the publication themselves.

## Results and discussion

### Detection of airborne algal and cyanobacterial taxa

**Fig 2:**
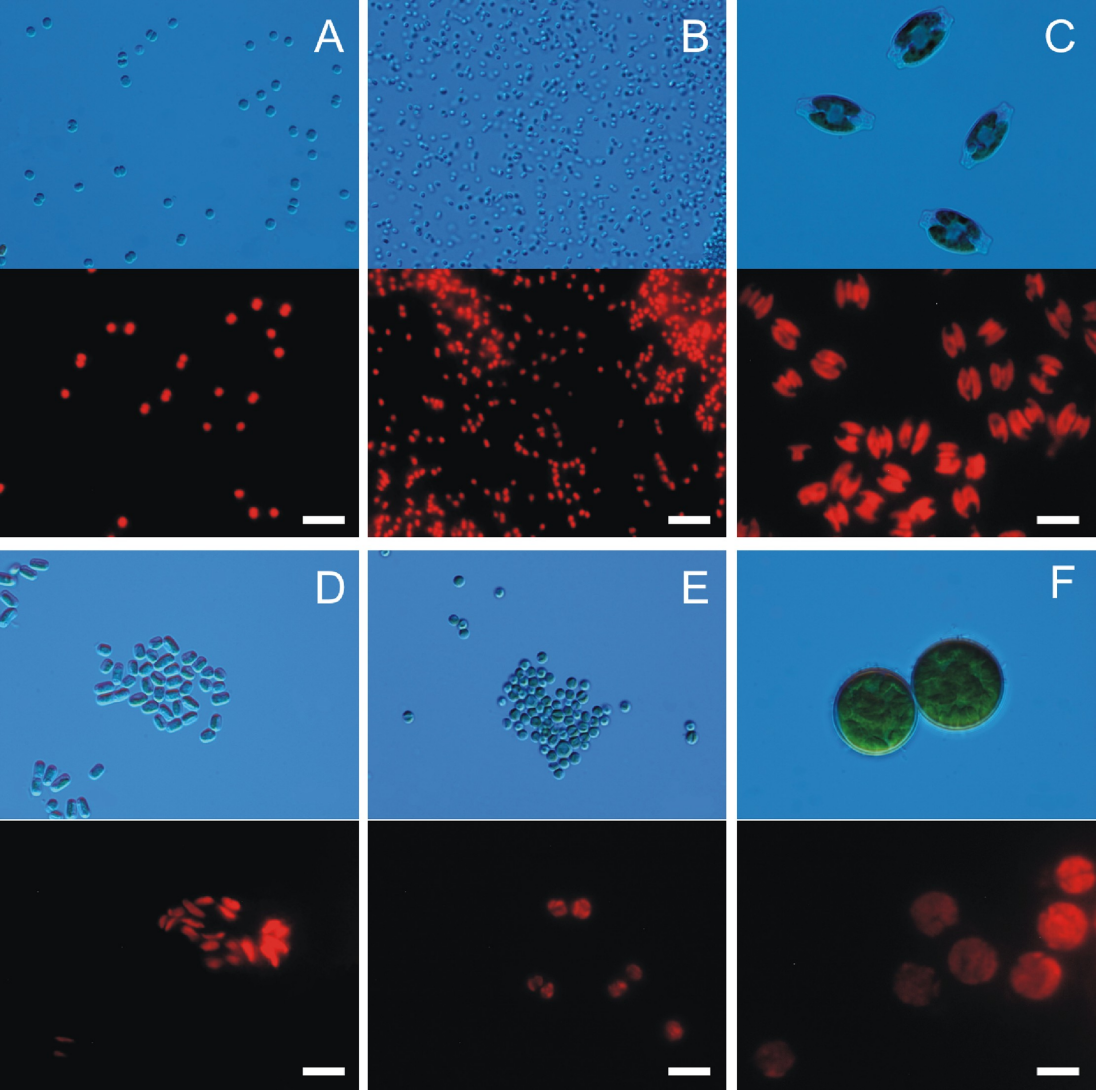
Examples of airborne cyanobacteria and microalgae obtained from the Adriatic Sea region that may induce negative health effects when inhaled. *Synechocystis* sp. (A), *Synechococcus* sp. (B), *Amphora* sp. (C), *Stichococcus* sp. (D), *Chlorella* sp. (E), *Chlorococcum* sp. (F), Scale bars = 10 μm.

To date, no studies have documented airborne algae and cyanobacteria in the West Basin of the northern Mediterranean Sea region. Our measurements are the first to be carried out in this area. They allowed identification of 15 taxa in total at 4 research stations during just four days. Among detected airborne algae and cyanobacteria, organisms that may induce negative health effects have been found (Table 1). In addition, potentially toxic species recorded in the study area were the most frequent and constituted 80% of all identified organisms. Among others, *Synechocystis* sp., *Chlorella* sp., *Chlorococcum* sp., *Stichococcus* sp., and *Amphora* sp. were identified (Fig 2).

**Table 1:** Airborne cyanobacteria and microalgae identified in the present study.

Station number	Latitude	Longitude	Collected phylum	Collected taxa
1	44º43’57”N	12º14’35”E	Cyanophyta	*Synechococcus* sp.
				*Synechocystis* sp.
			Chlorophyta	*Chlorella* sp.
				*Chlorococcum* sp.
			Bacillariophyta	Not detected
2	44º48’48”N	13º56’06”E	Cyanophyta	*Chroococcus* cf. *pulcherrimus*
			Chlorophyta	*Stichococcus* sp.
			Bacillariophyta	Not detected
3	42º27’10”N	18º34’03”E	Cyanophyta	*Gloeocapsa* sp.
				*Leptolyngbya* sp.
				*Woronichinia* sp.
			Chlorophyta	*Bracteacoccus* sp.
				*Stichococcus* sp.
				*Tetrastrum* cf. *heteracantum*
			Bacillariophyta	*Amphora* sp.
				*Licmophora* sp.
4	42º14’04”N	18º54’05”E	Cyanophyta	*Snowella* sp.
				*Synechocystis* cf. *salina*
			Chlorophyta	*Chlorella* sp.
				*Chlorococcum* sp.
			Bacillariophyta	*Amphora* sp.

In the first two stations, only cyanobacteria and microalgae occurred. This may be due to the strong and almost permanent blooms of picocyanobacteria recorded in the Italian waters, near which these stations are located [38–40]. As reported in the literature picocyanobacteria blooms may cause severe impoverishment of other microalgae species. As a consequence these organisms could occur more often in the air above the first two stations. In turn, at stations 3 and 4, which are located furthest from Italy we noted the highest species diversity. It may confirm the hypothesis that picocyanobacteria, especially during summer blooms, are able to limit the occurrence of competing algae, e.g., diatoms in water. Thus the presence of these algae in aerosols is getting to be lower [38–39].

**Fig 3:**
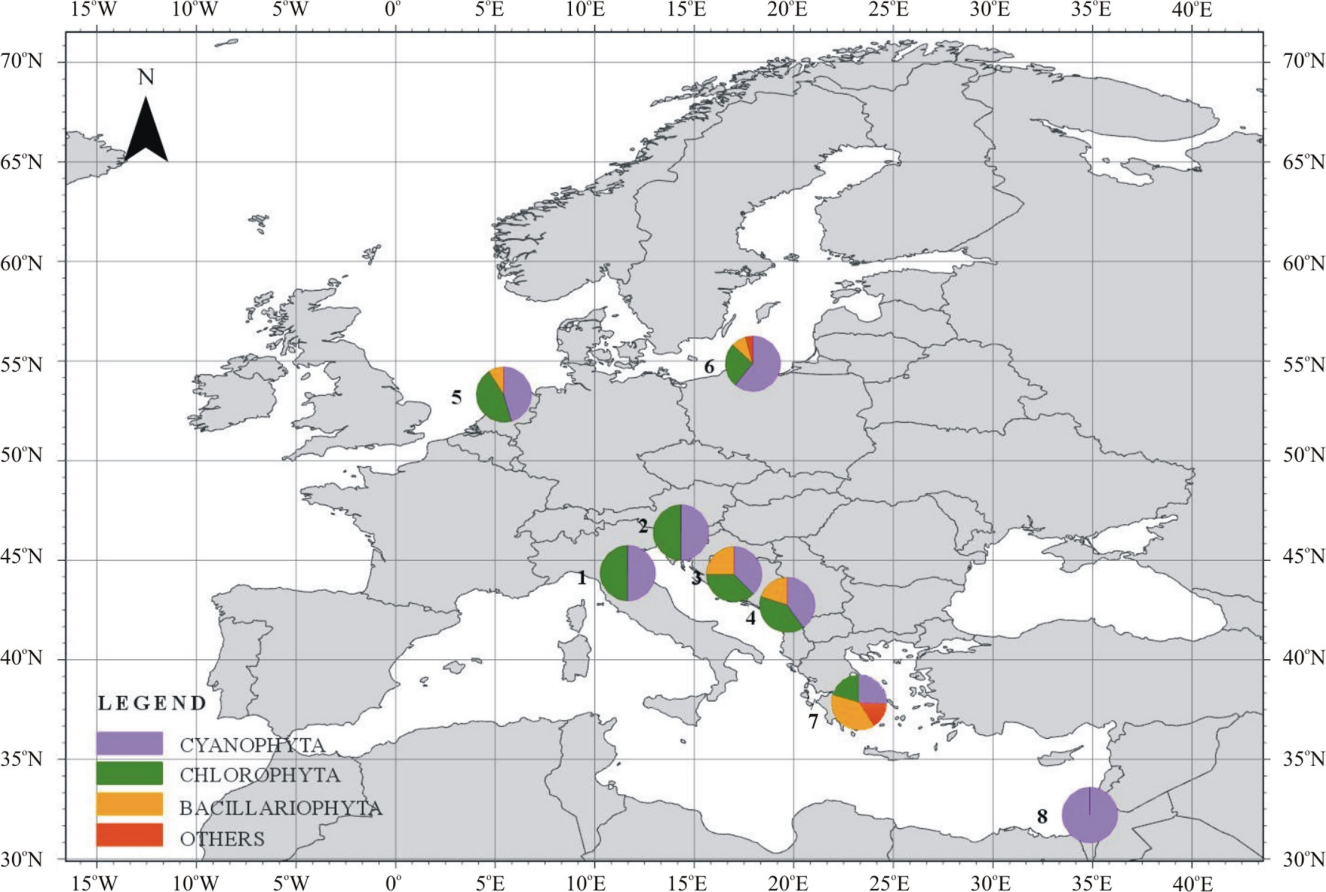
The percentage of individual taxa of airborne cyanobacteria and microalgae shared in the air at research stations and in the vicinity of the examined area (ArcGIS PRO 2.3.2). The stations were located in the northern part of Italy (station 1), Croatia (station 2), Montenegro (stations 3and 4), The Netherlands (station 5), northern part of Poland (station 6), Greece (station 7), and northern part of Egypt (station 8).

Despite the short sampling window (30 minutes), the number of identified taxa was relatively large, suggesting that many taxa remain to be documented. By comparison, the same number of airborne cyanobacteria and microalgae (15 taxa) were recorded in Egypt (Fig 3, station 8) during a one year sampling period, from 2004 to 2005 [41]. However, in this case the time of plates exposure was short (5 to 10 minutes). In New England (USA), Lee and Eggleston [24] also detected similar number of different airborne microalgal taxa: 5 Chlorophyta, 4 Bacillariophyta and 2 Cyanophyta. The authors employed various methods, such as exposing Petri dishes for 30 minutes, exposing bottles filled with liquid medium for 10 minutes and dipping Millipore prefilters into media. The largest number of taxa, numbering 62, was identified by Brown et al. [42] in Texas during campaign which took place in the 60s of the twentieth century. Researchers employed several different sampling techniques, e.g., Petri dishes exposed in flight and in a moving automobile. The results indicated that the method using deposition in Petri dishes gives comparable results regarding the number of collected taxa to the method using the mobile. Critical conditions in this case turned out to be a wind speed higher than 25 km/h or the duration of measurements (10–12h), taking place especially after strong winds [42].

Although the taxonomic compositions of cyanobacteria and microalgae vary worldwide, the biogeography of these airborne photosynthetic organisms is still poorly explored [5, 6, 43]. Some authors have showed that Cyanophyta dominate in tropical regions [4, 11, 44–46], whereas, Chlorophyta has been proved to be dominant in temperate regions [44, 47]. On the other hand, studies conducted by Lewandowska et al. [16] in the Baltic Sea coastal zone have shown that the presence of individual phyla is closely tied to the season. Nevertheless, there is still a need for more detailed research on cyanobacteria and microalgae in coastal regions of Europe, especially in tourist destinations [5]. There are just few reports in the literature on the taxa present in water during blooms in this regions [22, 48] and there is no information at all about their presence in the air at that time.

Our measurements indicated that Cyanophyta were most common among all identified taxa in the Adriatic Sea region (constituting the Northern Mediterranean area). They constituted 53% of all collected organisms, while Chlorophyta and Bacillariophyta accounted for 34% and 13%, respectively. However, when the occurrence of individual taxa at each station was analyzed separately, the relative proportion of Cyanophyta and Chlorophyta were the same only at stations 1 and 2 (Fig 3). At others two stations, diatoms were detected in addition to Cyanophyta and Chlorophyta. Bacillariophyta accounted for 25% and 20% of all taxa, respectively, at stations 3 and 4 (Fig 3). The taxonomic composition that we documented differs from that documented by Genitsaris et al. [9] within the Eastern Mediterranean (Fig 3, station 7). Researchers identified 59 taxa in 93 samples collected throughout one year (from August 2007 to November 2008) in Thessaloniki, Greece. In contrast to the results of our study, among the airborne cyanobacteria and microalgae, Bacillariophyta dominated, accounting for 42% of taxa (Fig 3, station 7). Additionally, Cyanophyta constituted only 20% and Chlorophyta 25%. Such pronounced differences noted in the region of the same sea but other basins can confirm that the presence of cyanobacteria and microalgae in the air is closely tied to local conditions. The biological variability of the waterbody can be influenced by, among others, seafloor depth and nutrient supply along with river runoff. An example of such waters is the northern part of the Adriatic Sea [49]. Other example of local conditions influence is Kastela Bay (Croatia), characterized by conditions particularly favourable for phytoplankton proliferation [50, 51].

Furthermore, the results obtained in our study are similar to those obtained from April to November 2015 in the southern Baltic Sea region by Lewandowska et al. [16] (Fig 3, station 6). The study included 120 samples, 42 from a marine station and 78 from a terrestrial station. Among the identified taxa, 41 cyanobacteria and microalgae were identified. Cyanophyta (49%), and Chlorophyta (48%) constituted the largest proportion of all presented airborne microorganisms. Lewandowska et al. [16] documented also one taxon, *Nannochloropsis* sp. from the phylum Ochrophyta, that has rarely been documented in the air by other scientists. This suggests that research on airborne cyanobacteria and microalgae being conducted in various regions of the world contributed to our understanding of the sporadic occurrence of these microbes in the air.

The tendency that Cyanophyta and Chlorophyta dominate in the air seems to be preserved in many parts of the world. Already at the beginning of the 20th century, pioneering studies conducted by van Overeem [52] in the Netherlands indicated that Cyanophyta and Chlorophyta constituted 46% and Bacillariophyta only 9% of all 11 identified taxa (Fig 3, station 5). Research conducted so far indicated also that in various parts of the world the only phylum noted in the air were Cyanophyta [41] (Fig 3, station 8). In these studies, researchers have, however, tended to exclusively focus on cyanobacteria and chemical pollution in the air. Thus, it is unclear whether microalgae were actually absent in some regions of the world or whether their absence originated from the lack of sufficient sampling or analytical effort. However, it can be stated that airborne cyanobacteria and microalgae originating from the Adriatic Sea region have been noted by several researchers in other parts of the world, except the cyanobacterium *Snowella* sp. and the green alga *Tetrastrum* sp., that had not been previously documented from atmospheric samples (Table 2).

**Table 2:** Cyanobacteria and microalgae originated from the Adriatic Sea region by other researchers noted in the air of various world locations.

Airborne microalgae and cyanobacteria	Sampling points locations	References
**Cyanophyta**		
*Gloeocapsa* sp.	India	[11]
	Poland	[16]
	Taiwan	[53]
*Leptolyngbya* sp.	Hawaii United States	[25]
	Malaysia	[46]
	Poland	[16]
*Snowella* sp.	Noted for the first time	
*Synechococcus* sp	India	[11]
	Poland	[16]
	United States	[42]
*Synechocystis* sp.	Hawaii United States	[54]
	India	[11]
	Poland	[16]
*Woronichinia* sp.	Poland	[16]
**Chlorophyta**		
*Bracteacoccus* sp.	Hawaii United States	[55]
	Taiwan	[53]
	United States	[47]
	United States	[56]
*Chlorella* sp.	Greece	[9]
	Hawaii United States	[55]
	Hawaii United States	[54]
	India	[11]
	Malaysia	[57]
	Mexico	[10]
	Nederland	[52]
	Poland	[16]
	Taiwan	[53]
	United States	[47]
	United States	[42]
	United States	[56]
	United States	[24]
*Chlorococcum* sp.	Hawaii United States	[55]
	Hawaii United States	[54]
	India	[11]
	India	[4]
	Malaysia	[57]
	Mexico	[10]
	Poland	[16]
	Taiwan	[53]
	United States	[47]
	United States	[42]
	United States	[24]
	United States	[56]
*Stichococcus* sp.	Hawaii United States	[55]
	India	[11]
	Nederland	[48]
	Poland	[16]
	United States	[47]
	United States	[42]
	United States	[24]
*Tetrastrum* sp.	Noted for the first time	
**Bacillariophyta**		
*Amphora* sp.	Greece	[9]
*Licmophora* sp	Greece	[9]

### Meteorological factors

Meteorological conditions can affect both the abundance of airborne microalgae and cyanobacteria as well as their species richness [16, 45, 54]. Given that our measurements were mobile, we do not have meteorological data from the sampling points. However, at present, there is a number of possibilities to use tools such as mathematical models or websites of scientific units with reliable meteorological results from the research area. One of such a tool is HYSPLIT model (http://www.arl.noaa.gov/ready.html) [58]. In our case we used this model to identify the origin of airborne cyanobacteria and microalgae over all measurement stations. It was very important to us to determine whether the identified species of cyanobacteria and microalgae had their source on land or in the sea. HYSPLIT model allows such an interpretation. The only one condition to remember is that bioaerosols should not be taken only under sea advection. Although air mass trajectory analysis indicate rather potential sources of pollutants it is commonly used to attempt to interpret results, including bioaerosols [1, 14, 16, 25, 36, 37, 59]. There is high demand on such an information which are important for the further determination of biogeography of cyanobacteria and microalgae present in the air [5].

**Fig 4:**
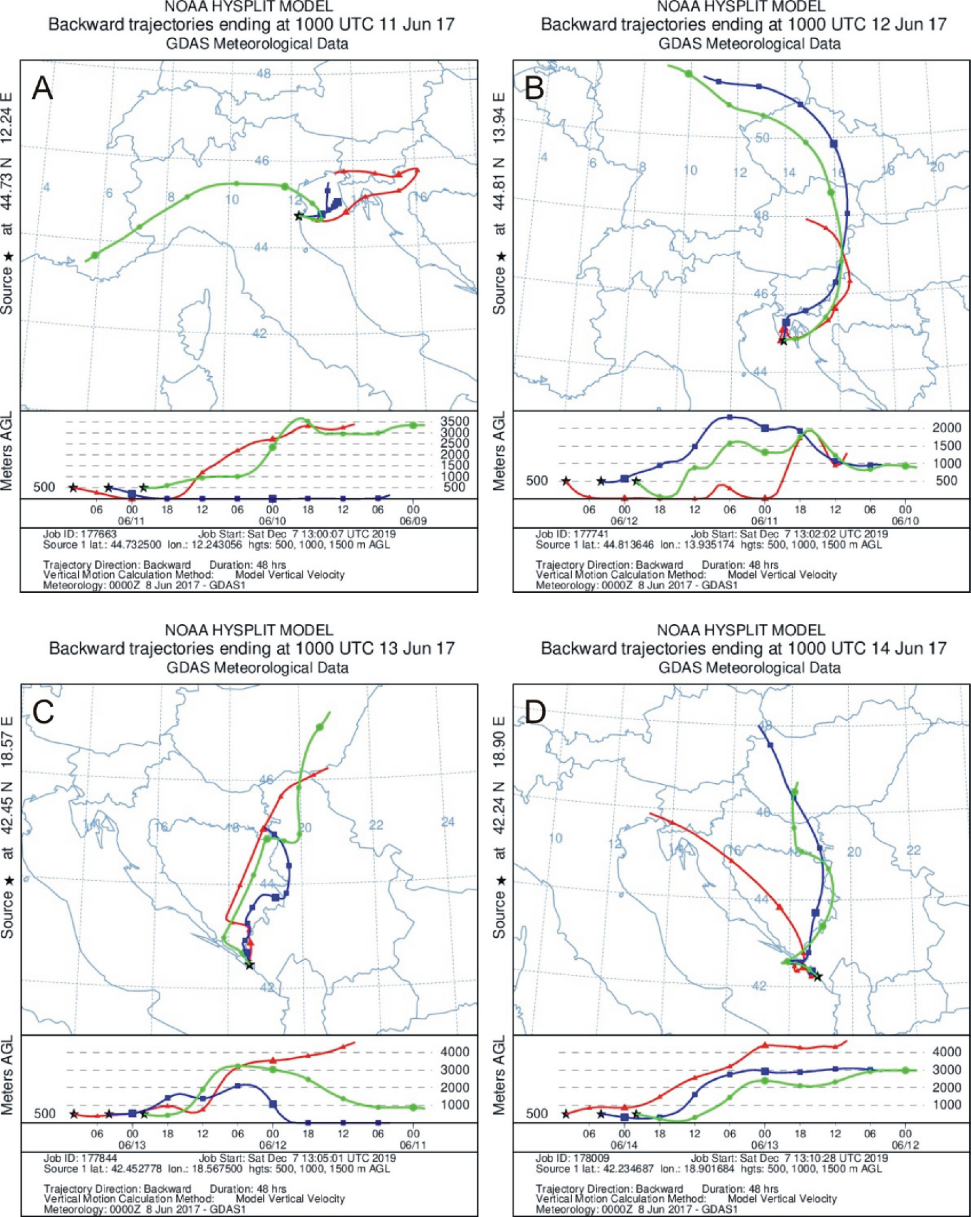
48-h air mass backward trajectory analysis (HYSPLIT) for 1^st^ (A), 2^nd^ (B), 3^rd^ (C), and 4^th^ (D) sampling station.

Air mass trajectories were determined for all research stations (Fig 4). The station 1, located in Italy was the only one (Fig 1), where we noted air masses from both the seaside and land (Fig 4A). The remaining stations were only under air masses from the mainland (Fig 4B–4D). This could have been important for species diversity of cyanobacteria and microalgae in the air (Table 1). At station 1, located in Italy on which mixed air masses were noted we identified species of cyanobacteria and common green algae occurring in both marine and terrestrial environments [60], i.e. *Chlorella* sp. and *Synechococcus* sp. (Table 1). Assuming that the efficiency of cyanobacteria and microalgae emission from the sea surface is analogous to the efficiency of spume drops effectively tearing off a wave, the wind speed recommended for this process is between 18.0 km/h and 39.6 km/h [61]. At the first measuring station, the average wind speed was only 12.6 km/h and was not sufficient to generate marine aerosols and transfer algae to the atmosphere. However, with a maximum value of 22.2 km/h, this process could already be effective and marine species could be present in the air. On the other hand such a wind speed is high enough for atmospheric transport of aerosols in regional scale and could be responsible for the presence of terrestrial microorganisms over the first station. Lewandowska and Falkowska [62] also pointed out that intensity of generating marine aerosols and aerosols transportation with air masses increase exponentially with wind speed higher than18 km/h over land and higher than 10 km/h over the sea. The process is the most noticeable with marine advection or/and under sea breeze [62].

At the remaining three stations, located in Croatia and Montenegro (Fig 1) land originated air masses transported from the north of the continent dominated (Fig 4B–4D). However, the point of origin for the air masses differed for every station. Hence the differences in obtained species composition between stations seem obvious (Table 1). The highest species diversity of the microorganisms was recorded at the third station, in Montenegro (Table 1), over which air masses were transported from above the border between Bosnia and Herzegovina and Croatia (Fig 4C). At this station, 8 taxa representing both cyanobacteria, green algae and diatoms were noted (Table 1). Even the wind speed was the lowest compared to other stations, on average equal to 4 km/h, its maximum value reached 23.0 km/h, what was enough for bioaerosol transportation with air masses [62]. The smallest number of taxa was recorded at station 2, located in Croatia. In this case the air mass trajectories indicate that overland masses originated in Central Europe and passed through the most areas in a certain time (Fig 4B). This means that the size of the area through which air masses flow is not a key factor which affect the number of taxa recorded. In addition at the second station the average wind speed was twice as high as at the third station, where the highest species diversity was recorded (S1 Table). Thus, wind speed probably is also not the main factor responsible for the aerosol biodiversity. Similar conclusions were reached by Schlichting [56], who determined the highest number of cyanobacteria and microalgae at lower wind speed. Probably the diversity of taxa results rather from the overlap of many factors at the same time. There are individual studies determining the impact of meteorological factor on the number of identified taxa as the most important parameter [5, 45]. However the number of cyanobacteria and microalgae in the seawater is not less important. Fraction of individual sea spray aerosol particles containing biological material increases with particle diameter (greater than 0.5 μm) and is impacted by phytoplankton blooms [63]. Emission rates for the natural bioaerosols mentioned here may hinge on their concentration in the source area [5]. Therefore, in the case of blooms of picocyanobacteria in water, their transport to the air is very possible. In addition, these organisms are lighter than e.g. diatoms, for which due to having heavy cell walls saturated with silica, wind speed will be of great importance [64]. As can be seen in this study, the amount of diatoms was relatively small. To confirm the above-mentioned theses, it is worth conducting more research in this area. So far, this type of research has not been conducted in this region, which is why this work particularly emphasizes their necessity.

In contrast to the role of wind speed, air mass trajectories allowed us to indicate potential sources of organisms such as *Tetrastrum* sp. and *Snowella* sp., which were recorded in air samples for the first time. At the third station, we found an interesting species in the air, *Tetrastrum* sp., which is typical for fresh waters [60]. This taxa had not been previously documented in the atmosphere over the Adriatic Sea by other researchers. In our measurements *Tetrastrum* sp. was listed only once and only in one sample. However, *Tetrastrum* sp. is indicated as one of the main greens in the Sava River flowing on the border between Bosnia and Herzegovina and Croatia [65]. So, during our measurements air masses, transported at a height of 500 m AGL, from over the Sava River could be responsible for *Tetrastrum* sp. presence in the air over the third station (Fig 4C). In general, in inland waters have been identified more species of phytoplankton organisms than in saltwater, which may explain their presence in the air, especially when air masses origin from the land [66]. Land air masses also flowed through station 4 in Montenegro. At this station *Snowella* sp. was found in the air for the first time. This species is characteristic for freshwater and slightly brackish reservoirs [60]. *Snowella* sp. has been also demonstrated in Mediterranean lakes [67, 68]. Obtained air mass trajectories confirmed that this organism could be carried to the investigated station from these lakes (Fig 4D).

### Potential impacts on human health

It is indicated that toxins produced by microalgae and cyanobacteria are being spread to the atmosphere and can be transported at least few kilometers from the coastal shoreline [69]. Furthermore, it is said that nasally applied microalgae toxins appears to have a 10-fold higher availability and toxicity than other ingestions [70]. Research conducted so far have shown that all organisms documented in the atmosphere, except for two green algae (*Bracteacoccus* sp. and *Tetrastrum* sp.), are capable of producing toxins or other harmful secondary metabolites [20, 71–82] (Table 3). Of these taxa, a particularly wide range of secondary metabolites have been documented for picocyanobacteria. Detailed information on the harmful secondary metabolites of the picocyanobacteria *Synechococcus* sp. and *Synechocystis* sp. has been provided earlier by Śliwińska-Wilczewska et al. [83].

**Table 3:** Examples of toxins and other harmful compounds detected from cyanobacteria and microalgae.

Collected phylum	Collected taxa	Compound	References
Cyanophyta	*Chroococcus* sp.	LPS	[20]
	*Gloeocapsa* sp.	LPS, MC	[20, 71]
	*Leptolyngbya* sp.	coibamide A, crossbyanols A−D, LPS, MC	[20, 72, 73]
	*Snowella* sp.	LPS, MC	[20, 74]
	*Synechococcus* sp.	BMAA, fatty acids, geosmin, hemolysins, linolenic acid, lipopeptide, LPS, MC, MIB, synechobactins A–C, thionsulfolipid	[20, 70, 72, 75]
	*Synechocystis* sp.	BMAA, anatoxin-a, fatty acid, LPS, MC, triterpenoid	[20, 70, 74, 75]
	*Woronichinia* sp.	anatoxin-a, LPS	[20, 74]
Chlorophyta	*Bracteacoccus* sp.	-	
	*Chlorella* sp.	chiorellin, MAA, polyamines	[76, 77]
	*Chlorococcum* sp.	alkaloids, aminoacids, carbohydrates, fatty acids, favanoids, saponins	[78]
	*Stichococcus* sp.	fatty acids, MAA, thiol peptides	[79, 76, 77]
	*Tetrastrum* sp.	-	
Bacillariophyta	*Amphora* sp.	domoic acid	[80, 81]
	*Licmophora* sp.	unidentified allelochemicals	[82]

BMAA–*β*-N-methylamino-L-alanine, MAA–mycosporine-like amino acids, MC–microcystin, MIB– 2-methylisoborneol, LPS–lipopolysaccharides

The negative impact of airborne cyanobacteria and microalgae on human health is caused by their inhalation [5, 9, 12, 15, 16]. Depending on their size, cyanobacteria and microalgae can settle in different sections of the human respiratory tract [5, 84]. Facciponte and coauthors [84] identified Cyanobacteria that infiltrate not only the upper respiratory tract (nasal cavity), but also the central airway (lung: right upper lobe) suggesting that aerosolization may be a significant route of human exposure. It has been estimated that humans inhale approximately 1500 algal cells per day [25], however, it is still unclear how much microorganisms should be inhaled to cause adverse health effects. Inhalation of airborne cyanobacteria and microalgae can lead to allergies, rhinitis, asthma, bronchitis, and dermatitis [9, 12]. Of the taxa identified in this study, inhalation of *Synechocystis* sp., *Synechococcus* sp., *Bracteacoccus* sp., *Chlorella* sp., *Chlorococcum* sp., *Stichococcus* sp., and *Amphora* sp. is considered most harmful to humans according to Genitsaris et al. [9]. Although *Woronichinia* sp., detected by Lewandowska et al. [16] and *Tetrastrum* sp. as well as *Snowella* sp. detected in this study do not appear in the list of detected airborne algae of Genitsaris et al. [9]. That is why it is unclear whether inhalation of these taxa is harmful to humans.

Even some reports on the toxicity of the aforementioned cyanobacteria and microalgae already exist; they are primarily concerned with their impact on the aquatic environment. Thus, much still remains to be learned on the potential harmful effects, especially of newly detected airborne cyanobacteria and microalgae. Given the potential harm they could pose, the list of harmful taxa made by Genitsaris et al. [9] should be updated.

## Conclusions

Research conducted in the Adriatic Sea region showed the presence of cyanobacteria and microalgae in atmospheric air. This research was conducted over a relatively short period of time, and the prevailing meteorological conditions did not favor the transport of microorganisms from above water to land. For such a short sampling time, the number of identified taxa was considerable compared to records from the literature. Our findings suggest that beaches can have negative impacts on human health, even if people refrain from entering the water. Specifically, we show that among the 15 taxa identified in the Adriatic Sea region, inhalation of at least 8 genera of airborne cyanobacteria and microalgae can have adverse effects on human health. In addition, except for two green algal taxa, all the identified organisms are capable of producing harmful metabolites, including toxins. Moreover, we documented the presence of *Snowella* sp. and *Tetrastrum* sp., taxa that had not been previously identified in other studies of airborne cyanobacteria and microalgae. Additional taxonomic, molecular and toxicological studies are needed to more fully characterize the composition and environmental roles of airborne cyanobacteria and microalgae. Our results demonstrate the promise of the Adriatic Sea region as a site for future research on airborne cyanobacteria and microalgae especially in the context of their harmful impacts on human health.

## Supporting information

S1 Table The locations of stations, country of origin, times of sample collection, and environmental conditions during the sampling period. Meteorological data comes from http://www.ogimet.com/.
(DOC)
