# Supplementary material for: The first characterization of airborne cyanobacteria and microalgae in the Adriatic Sea region
Source: PLoS One. 2020 Sep 10;15(9):e0238808. doi: 10.1371/journal.pone.0238808 (PMC7482968; doi:10.1371/journal.pone.0238808)
Supplement: S1 Table — Meteorological data comes from http://www.ogimet.com/. (DOC) [file pone.0238808.s001.doc]

**S1 Table.**

**The locations of measurement stations, country of origin, times of sample collection, and environmental conditions during the sampling period.**

| **Station** | **Country** | **Location** | **Date** | **Altitude** | **Salinity** | **pH** | **T_air_ [°C]** | **Rh [%]** | **V [km h^−1^]**  x̅ max | |
| --- | --- | --- | --- | --- | --- | --- | --- | --- | --- | --- |
| 1 | Italy | 44º43’57”N  12º14’35”E | 11.06.2017 | 0.3 | 23.5 | 8.25 | 23.9 | 58.1 | 12.6 | 22.2 |
| 2 | Croatia | 44º48’48”N  13º56’06”E | 12.06.2017 | 5.9 | 36.5 | 8.12 | 22.6 | 51.7 | 8.0 | 14.9 |
| 3 | Montenegro | 42º27’10”N 18º34’03”E | 13.06.2017 | 17.1 | 33.7 | 8.18 | 22.7 | 65.5 | 4.0 | 23.0 |
| 4 | Montenegro | 42º14’04”N 18º54’05”E | 15.06.2017 | 59.2 | 36.1 | 8.17 | 24.9 | 76.3 | 11.0 | 18.0 |

where: T_air_ – mean air temperature [°C], Rh - relative humidity [%], Vx̅ – average wind speed [km h^−1^], V_max_  maximal wind speed

Air temperature, relative humidity and wind speeds comes from https://www.ogimet.com
